# Causal relationship between gut microbiota and tuberculosis: a bidirectional two-sample Mendelian randomization analysis

**DOI:** 10.1186/s12931-023-02652-7

**Published:** 2024-01-04

**Authors:** Zongxiang Yuan, Yiwen Kang, Chuye Mo, Shihui Huang, Fang Qin, Junhan Zhang, Fengyi Wang, Junjun Jiang, Xiaoxiang Yang, Hao Liang, Li Ye

**Affiliations:** 1 Guangxi Key Laboratory of AIDS Prevention and Treatment, School of Public Health, Guangxi Medical University, Nanning, 530021 China; 2 Collaborative Innovation Centre of Regenerative Medicine and Medical BioResource Development and Application Co-Constructed by the Province and Ministry, Life Science Institute, Guangxi Medical University, Nanning, 530021 Guangxi China; 3 Department of Infectious Diseases in Children, Maternity and Child Health Care of Guangxi Zhuang Autonomous Region, Nanning, 530003 Guangxi China

**Keywords:** Gut microbiota, Tuberculosis, Respiratory tuberculosis, Extrapulmonary tuberculosis, Mendelian randomization, Causal relationship

## Abstract

**Background:**

Growing evidence from observational studies and clinical trials suggests that the gut microbiota is associated with tuberculosis (TB). However, it is unclear whether any causal relationship exists between them and whether causality is bidirectional.

**Methods:**

A bidirectional two-sample Mendelian randomization (MR) analysis was performed. The genome-wide association study (GWAS) summary statistics of gut microbiota were obtained from the MiBioGen consortium, while the GWAS summary statistics of TB and its specific phenotypes [respiratory tuberculosis (RTB) and extrapulmonary tuberculosis (EPTB)] were retrieved from the UK Biobank and the FinnGen consortium. And 195 bacterial taxa from phylum to genus were analyzed. Inverse variance weighted (IVW), MR-Egger regression, maximum likelihood (ML), weighted median, and weighted mode methods were applied to the MR analysis. The robustness of causal estimation was tested using the heterogeneity test, horizontal pleiotropy test, and leave-one-out method.

**Results:**

In the UK Biobank database, we found that 11 bacterial taxa had potential causal effects on TB. Three bacterial taxa *genus.Akkermansia*, *family.Verrucomicrobiacea*, *order.Verrucomicrobiales* were validated in the FinnGen database. Based on the results in the FinnGen database, the present study found significant differences in the characteristics of gut microbial distribution between RTB and EPTB. Four bacterial taxa* genus.LachnospiraceaeUCG010*, *genus.Parabacteroides*, *genus.RuminococcaceaeUCG011*, and *order.Bacillales* were common traits in relation to both RTB and TB, among which *order.Bacillales* showed a protective effect. Additionally, *family.Bacteroidacea* and *genus.Bacteroides* were identified as common traits in relation to both EPTB and TB, positively associating with a higher risk of EPTB. In reverse MR analysis, no causal association was identified. No significant heterogeneity of instrumental variables (IVs) or horizontal pleiotropy was found.

**Conclusion:**

Our study supports a one-way causal relationship between gut microbiota and TB, with gut microbiota having a causal effect on TB. The identification of characteristic gut microbiota provides scientific insights for the potential application of the gut microbiota as a preventive, diagnostic, and therapeutic tool for TB.

**Supplementary Information:**

The online version contains supplementary material available at 10.1186/s12931-023-02652-7.

## Introduction

Tuberculosis (TB), caused by airborne *Mycobacterium tuberculosis* (*M.tuberculosis*), is one of the most common infectious diseases in the world [1]. Despite the fact that lung is the primary site of *M.tuberculosis* infection, more than 15% of TB cases worldwide arise as extrapulmonary infections that can invade any organ system [2]. Nowadays, TB continues to contribute significantly to global morbidity and mortality [3], resulting in over 1 million deaths annually [4, 5]. Numerous epidemiological studies have identified some potential immune, environmental, and host genetic predisposing variables related to the onset and progression of TB, such as poverty [5], age [6], malnutrition [7], diabetes [8], and immunodeficiency [4, 7]. However, the interactions between TB and gut microbiota have received limited attention.

The gut microbiota is considered the “forgotten organ” of the human body [9] and is involved in various physiological processes [10]. Previous studies have shown that gut microbiota significantly affects the host immune system [11, 12] and dysbiosis in microbiome composition may contribute to resistance or susceptibility to TB [13]. To elucidate this issue, many epidemiologic studies have been conducted, but the results are inconsistent. Some studies [14–17] have indicated patients with active TB, new TB, or recurrent TB exhibit reduced microbial diversity compared to healthy individuals. However, Luo et al. [18] found that higher gut microbial diversity in patients with new or recurrent TB. Furthermore, with regard to changes in specific genera of bacteria, the reported results are also inconsistent. For instance, Wang [14] and Ye et al. [15] reported an increase in *Bacteroidetes* and a decrease in *Actinobacteria*, *Bifidobacteriales*, and *Bifidobacteriaceae* in untreated patients with active TB, while Ding et al. [16] found the opposite results. In summary, the relationship between the gut microbiota and TB remains elusive, and whether there is a causal relationship as well as the direction of the causal relationship are unclear. The above results are based on observational studies, with the limitations in the susceptibility to confounding factors such as environment, age, dietary habits, co-morbid infections, and malnutrition. In addition, there are inherent difficulties in confirming the temporal relationship between exposure and outcome. Therefore, there is an urgent need to explore the causal relationship between the TB and gut microbiota with the more refined research approaches.

With the rapid increase in microbiota and genetic data of complex diseases, Mendelian randomization (MR) [19] has been widely used in recent years. The MR approach employs the genome-wide association studies (GWAS) to identify single nucleotide polymorphisms (SNPs) that act as instrumental variables (IVs), allowing for causal inferences between exposure variables and outcomes [20]. The MR process is based on the random allocation of alleles in Mendelian laws of inheritance, while avoiding the influence of confounding factors [19, 21]. Hence, we conducted a bidirectional two-sample MR analysis adopting summary statistics of large GWAS from the MiBioGen consortium, the UK Biobank, and the FinnGen consortium for the first time to assess the causal relationship between gut microbiota and TB phenotypes comprehensively.

## Methods

### Data sources

Gut microbiota data used for exposure variables were obtained from the MiBioGen consortium. The consortium had curated and analyzed genome-wide genotype and 16S fecal microbiome information for 18,340 individuals from 24 cohorts, the majority of whom had European ancestry (18 cohorts, n = 14,306) [22]. In addition, covariates such as sex and age were adjusted for all cohorts. We excluded 15 bacterial taxa without specific species names (3 unknown families and 12 unknown genera) and 1 duplicate bacterial taxon [23] (*class.Verrucomicrobia* and *order.Verrucomicrobiales*). As a result, 195 bacterial taxa (119 genera, 32 families, 20 orders, 15 classes, and 9 phyla) were finally included as exposures for subsequent MR analyses.

The GWAS summary data of TB was from two databases. TB cases in the UK Biobank database were identified as “self-reported diseases” and were classified in the GWAS catalog (GWAS ID: GCST90038710). The study [24] covered 484,598 participants, including 2473 TB infected individuals and 482,125 controls. Age, sex, BMI, assessment center, ethnicity, batch, and the first 20 principal components were corrected during analysis. Moreover, we obtained the TB risk-related dataset from the FinnGen consortium R9 release for validation, which included 2432 cases and 374,845 controls (https://www.finngen.fi/en). This data included two types of TB infection: respiratory tuberculosis (RTB) (1793 cases and 374,922 controls) and TB in other organs named extrapulmonary tuberculosis (EPTB) (676 cases and 374,845 controls). The individuals' sex, age, the first 10 principal components, and genotyping batch were also underwent correction.

### Instrumental variables (IVs)

The validity of MR analysis depends on three key assumptions [19, 25]: (1) IVs are closely related to exposure; (2) IVs are not associated with any confounding variables; (3) IVs affect outcome only through exposure.

We determined necessary IVs according to the above core principles and followed the STROBE-MR statement [26] (Additional file 1: Table S1). First, we fixed the significance level at *P* < 1.0 × 10^–5^ and selected a group of SNPs strongly associated to gut microbiota as IVs for identifying sufficient candidate instruments. In the reverse MR analysis, the significance level was set to *P* < 1.0 × 10^–5^ since no tuberculosis-associated SNPs reached the threshold of *P* < 5.0 × 10^–8^. Second, we used samples from the 1000 Genomes European Project as a reference, and applied clumping to restrict SNPs with low linkage disequilibrium (r^2^ < 0.001; genetic distance = 10,000 kb). Third, SNPs with minor allele frequency (MAF) ≤ 0.01 were removed. Subsequently, we harmonized the exposure and outcome data. When palindromic SNPs existed, the forward strand alleles were inferred based on allele frequency information. Finally, the PhenoScanner website [27] was searched to investigate whether the selected SNPs are associated with confounding traits (BMI, weight, smoking or tobacco, alcohol, and pulmonary function) at a significance level of 5.0 × 10^–8^ (http://www.phenoscanner.medschl.cam.ac.uk/).

### Statistical analysis

The F-statistic was used to assess the strength of IVs. If the corresponding F-statistic was much greater than 10, it was considered that there was small possibility of weak IVs bias. The detailed calculation formula [28] was as follows: ${\text{F}}={{\text{R}}}^{2}\times ({\text{N}}-2)/(1-{{\text{R}}}^{2})$, where N represented the sample size of the exposure data, R^2^ represented the proportion of variance in exposure explained by genetic variation. The R^2^ value was estimated using the formula [29]:

$${{\text{R}}}^{2}=2\times {\text{EAF}}\times \left(1-{\text{EAF}}\right)\times \frac{{{\text{beta}}}^{2}}{\left(2\times {\text{EAF}}\times \left(1-{\text{EAF}}\right)\times {{\text{beta}}}^{2}+2\times {\text{EAF}}\times \left(1-{\text{EAF}}\right)\times {\text{se}}\times {\text{N}}\times {{\text{beta}}}^{2}\right)},$$

Where EAF referred to the effect allele frequency (EAF) of the SNP, beta represented the estimated effect size of the SNP, and se denoted the standard error.

In this study, we performed a bidirectional MR Analysis, using a variety of methods to examine whether there was a causal relationship between the gut microbiota and TB. IVW was employed as the main method to assess causal effects. This method utilized information from all IVs and aggregated the effect sizes of multiple IVs into a single estimate, making the results precise and robust [30]. We also performed secondary analyses using MR-Egger regression [31], ML [32], weighted median [33], and weighted mode, to provide evidence of effectiveness under different conditions. If the forward MR analysis result was positive, the reverse MR analysis would be performed. To ensure the exposure was directionally causal for the outcome, we conducted the additional MR Steiger directionality test [34].

To assess the robustness of the significant results, several sensitivity analyses were performed. The potential influence of horizontal pleiotropy was evaluated by MR-Egger regression detection [35] and MR-PRESSO global test analysis [36]. MR-PRESSO outlier tests eliminated the effect of pleiotropy by removing outliers. Cochran’s IVW Q statistics were used to quantify the heterogeneity of IVs [37]. Meanwhile, leave-one-out analysis was performed to determine whether the causal association signal was driven by a single SNP. Furthermore, based on the Bonferroni correction method, we established statistical thresholds for multiple comparisons at each feature level (phylum: 0.05/9, class: 0.05/15, order: 0.05/20, family: 0.05/32, and genus: 0.05/119). A significant causal relationship existed when the IVW method produced a *P* value lower than the adjusted *P* value. Otherwise, it was considered suggestive evidence of a potential association.

All MR analyzes in this study were performed based on TwoSampleMR [34] (version 0.5.4) and MR-PRESSO [36] (version 1.0) packages in R software (version 4.2.2). Forestploter package was used to create forest plots (version 1.1.0), and circlize package [38] was utilized for drawing circular heat maps (version 0.4.15).

## Results

### IVs selection of gut microbiota

**Fig. 1:**
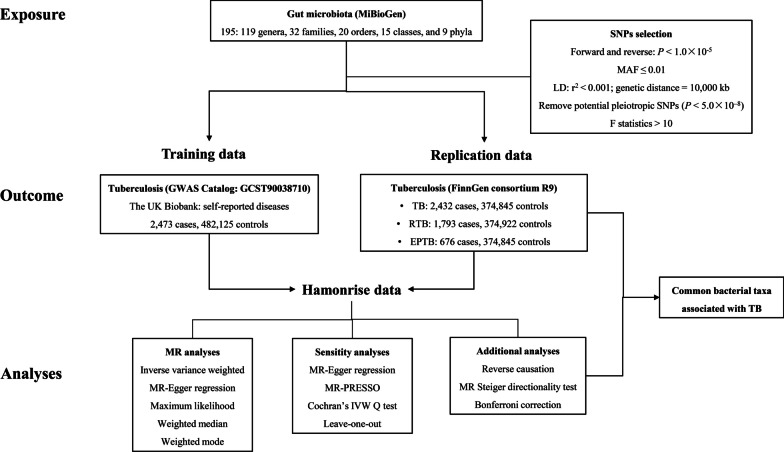
Research design and flow chart of this study. TB, tuberculosis; RTB, respiratory tuberculosis; EPTB, extrapulmonary tuberculosis; SNP, single nucleotide polymorphism; MAF, minor allele frequency; LD, linkage disequilibrium; IVW, inverse variance weighted; MR-PRESSO, Mendelian randomization pleiotropy residual sum and outlier

The flow chart of this study was shown in Fig. 1. To avoid potential pleiotropic effects caused by confounders, we removed potential confounding SNPs associated with these confounders of gut microbiota and TB by searching the PhenoScanner website (Additional file 1: Table S2). As a result, a total of 2,544 SNPs were identified as IVs for 195 bacterial taxa according to the above selection criteria. The F-statistic for IVs ranged from 10.781–95.389, indicating no evidence of weak instrumental bias. The details of these IVs in different databases were shown in Additional file 1: Table S3, S4.

### Causal inference between the gut microbiota and TB by in the UK Biobank database

**Fig. 2:**
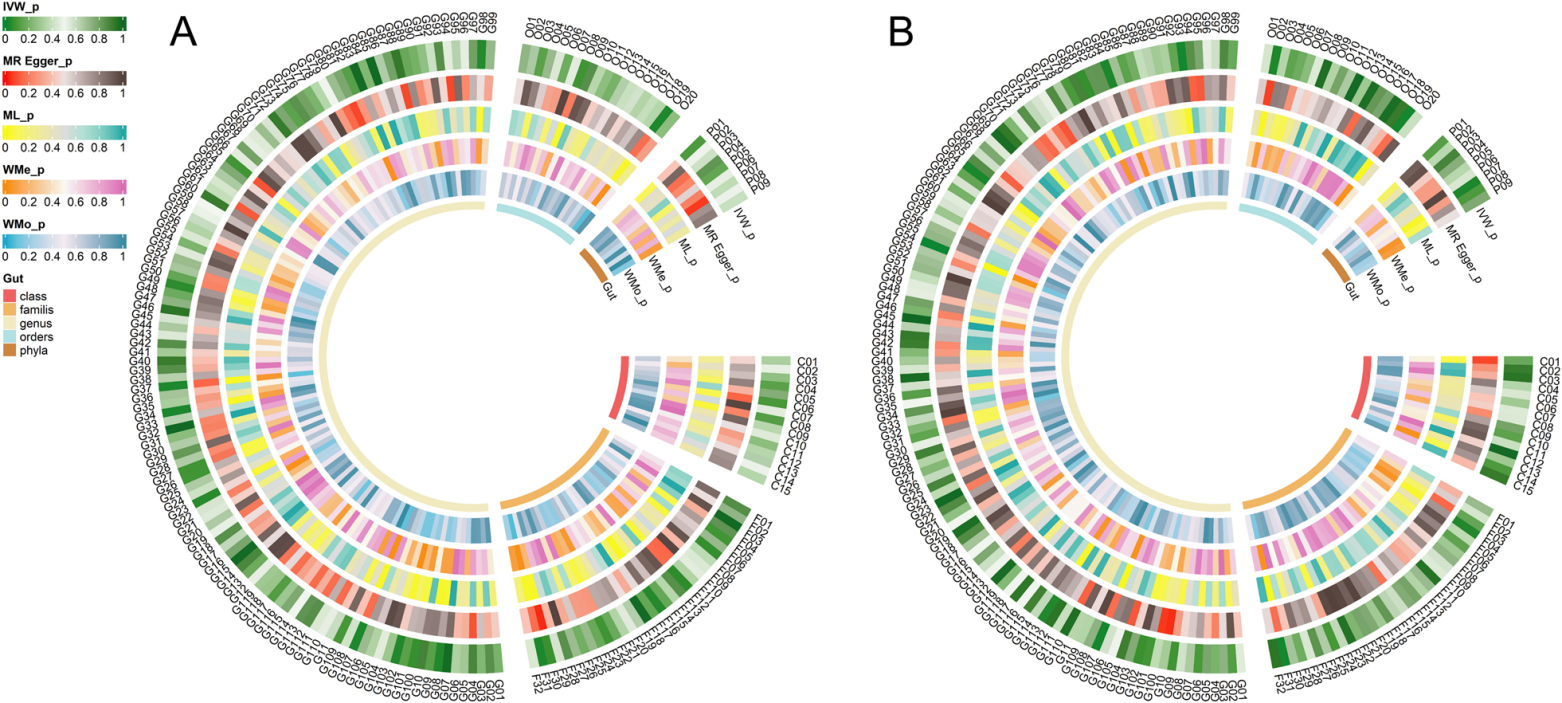
Causal effect of the gut microbiota on TB by MR analyses. **A** In the UK Biobank database; **B** In the FinnGen ddatabase. From outside to inside, the P values of IVW_*p*, ML_*p*, MR Egger_*p*, WMe_*p*, and WMo_*p* were represented, respectively. TB, tuberculosis; IVW, inverse variance weighted; ML, maximum likelihood; WMe, weighted median; WMo, weighted mode

**Fig. 3:**
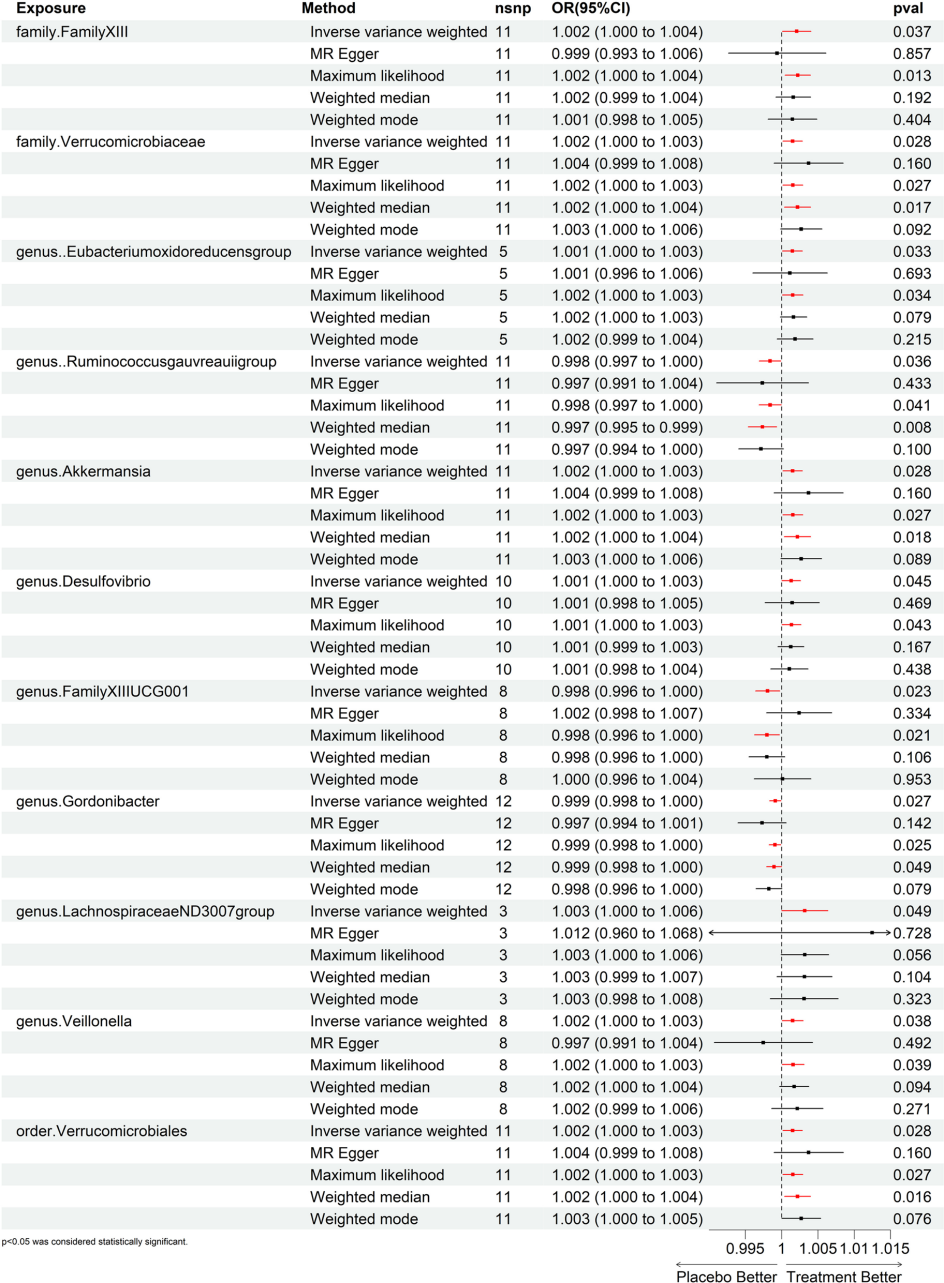
A forest plot of causal effect of gut microbiota on TB in the UK Biobank database (*P-*_*IVW*_ < 0.05). TB, tuberculosis; OR, odds ratio; CI, confidence interval

Then we performed MR analysis of TB dataset from the UK Biobank database. Figure 2 and Additional file 1: Table S5 presented the impact of changes in the abundance of 195 bacterial taxa on the risk of TB. According to the IVW analysis, 11 bacterial taxa of genetically predicted gut microbiota were found to be related to the risk of TB. As shown in Fig. 3, *family.FamilyXIII* (*OR* = 1.002, *95% CI* 1.000–1.004, *P* = 0.037), *family.Verrucomicrobiaceae* (*OR* = 1.002, *95% CI* 1.000–1.003, *P* = 0.028), *genus..Eubacteriumoxidoreducensgroup* (*OR* = 1.001, *95% CI* 1.000–1.003, *P* = 0.033), *genus.Akkermansia* (*OR* = 1.002, *95% CI* 1.000–1.003, *P* = 0.028), *genus.Desulfovibrio* (*OR* = 1.001, *95% CI* 1.000–1.003, *P* = 0.045), *genus.LachnospiraceaeND3007group* (*OR* = 1.003, *95% CI* 1.000–1.006, *P* = 0.049), *genus.Veillonella* (*OR* = 1.002, *95% CI* 1.000–1.003, *P* = 0.038), and *order.Verrucomicrobiales* (*OR* = 1.002, *95% CI* 1.000–1.003, *P* = 0.028) were suggestive associated with the increased risks of TB. Conversely, *genus*.*Ruminococcusgauvreauiigroup* (*OR* = 0.998, 95% *CI* 0.997–1.000, *P* = 0.036), *genus.FamilyXIIIUCG001* (*OR* = 0.998, 95% *CI* 0.996–1.000, *P* = 0.023), and *genus.Gordonibacter* (*OR* = 0.999, 95% *CI* 0.998–1.000, *P* = 0.027) exhibited suggestive protective effects against TB. Similar results were also obtained in ML analyses. Nevertheless, these associations were no longer significant after the Bonferroni correction.

### Causal inference between the gut microbiota and TB by in the FinnGen database

**Fig. 4:**
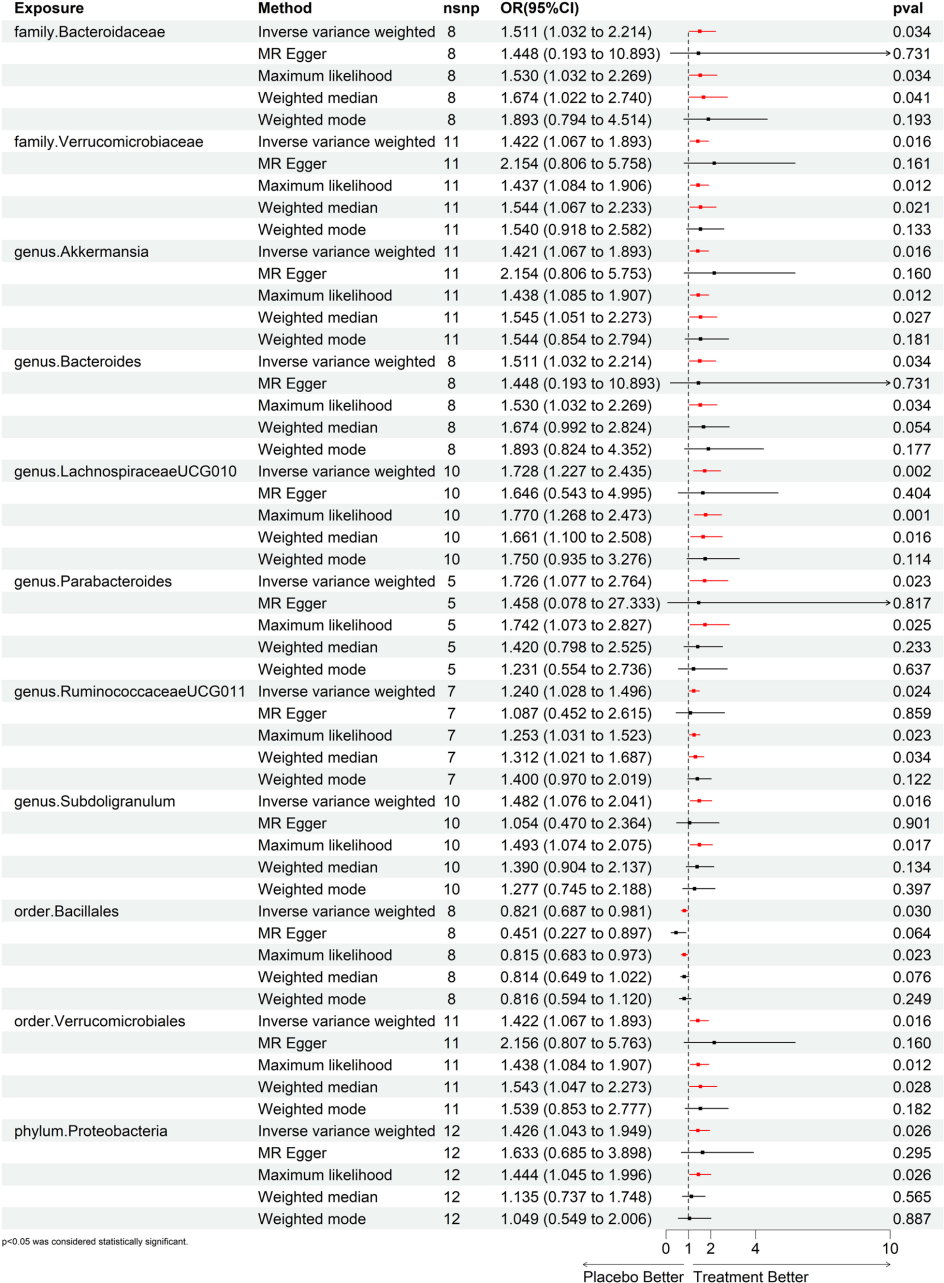
A forest plot of causal effect of gut microbiota on TB in the FinnGen database (*P-*_*IVW*_ < 0.05). TB, tuberculosis; OR, odds ratio; CI, confidence interval

To further explore the causal association between the gut microbiota and TB, we simultaneously performed MR analysis in another large-scale population-based biobank from Europe (the FinnGen database). The FinnGen database contains a large amount of shared DNA in linkage disequilibrium and is rich in alleles that are uncommon in other populations, and thus it is highly valuable in identifying rare single-gene disorders and the genetic variants responsible for them [39]. Figure 2 and Additional file 1: Table S6 presented the impact of changes in the abundance of 195 bacterial taxa on the risk of TB. The IVW analysis results were presented in Fig. 4, and we identified 11 bacterial taxa. Specifically, the risk of TB was increased by *family.Bacteroidaceae* (*OR* = 1.511, 95% *CI* 1.032–2.214, *P* = 0.034), *family.Verrucomicrobiaceae* (*OR* = 1.422, 95% *CI* 1.067–1.893, *P* = 0.016), *genus.Akkermansia* (*OR* = 1.421, 95% *CI* 1.067–1.893, *P* = 0.016), *genus.Bacteroides* (*OR* = 1.511, 95% *CI* 1.032–2.214, *P* = 0.034), *genus.LachnospiraceaeUCG010 (OR* = 1.728, 95% *CI* 1.227–2.435, *P* = 0.002), *genus.Parabacteroides* (*OR* = 1.726, 95% *CI* 1.077–2.764, *P* = 0.023), *genus.RuminococcaceaeUCG011* (*OR* = 1.240, 95% *CI* 1.028–1.496, *P* = 0.024), *genus.Subdoligranulum* (*OR* = 1.482, 95% *CI* 1.076–2.041, *P* = 0.016), *order.Verrucomicrobiales* (*OR* = 1.422, 95% *CI* 1.067–1.893, *P* = 0.016), *phylum.Proteobacteria* (*OR* = 1.426, 95% *CI* 1.043–1.949, *P* = 0.026). Conversely, the relative abundance of *order.Bacillales* (OR = 0.821, 95% CI 0.687–0.981, P = 0.030) showed a negative association with the risk of TB. Similar trends were observed in ML, MR-Egger, weighted median, and weighted mode methods. Nevertheless, these associations were no longer significant after the Bonferroni correction.

### Causal inference between the gut microbiota and RTB, EPTB in the FinnGen database

In this study, we also separately conducted MR analyses for the gut microbiota and two types of TB (RTB and EPTB) to establish a more compelling causal association between them. For RTB, the causal effects of 195 bacterial taxa on the risk of RTB were shown in the Additional file 2: Fig. S1 and Additional file 1: Table S7. As illustrated in Additional file 3: Fig. S2, the results of the IVW analysis showed that *genus..Eubacteriumbrachygroup* (*OR* = 1.329, 95% *CI* 1.084–1.630, *P* = 0.006), *genus.Eisenbergiella* (*OR* = 1.258, 95% *CI* 1.004–1.575, *P* = 0.046), *genus.LachnospiraceaeUCG010* (*OR* = 1.799, 95% *CI* 1.243–2.604, *P* = 0.002), *genus.Marvinbryantia* (*OR* = 1.437, 95% *CI* 1.009–2.048, *P* = 0.045), *genus.Parabacteroides* (*OR* = 1.765, 95% *CI* 1.020–3.053, *P* = 0.042), *genus.RuminococcaceaeUCG011* (*OR* = 1.245, 95% *CI* 1.002–1.549, *P* = 0.048) significantly increased the risk of RTB. In contrast, the decrease in RTB risk could attribute to the increase of genetically predicted *family.BacteroidalesS24.7group* (*OR* = 0.745, 95% *CI* 0.555–1.000, *P* = 0.050), *family.Peptococcaceae* (*OR* = 0.703, 95% *CI* 0.512–0.963, *P* = 0.028), *genus.Holdemania* (*OR* = 0.758, 95% *CI* 0.584–0.982, *P* = 0.036), *genus.RuminococcaceaeUCG005* (*OR* = 0.729, 95% *CI* 0.535–0.992, *P* = 0.045), *genus.Ruminococcus1* (*OR* = 0.673, 95% *CI* 0.454–1.000, *P* = 0.050), *order.Bacillales* (*OR* = 0.753, 95% *CI* 0.617–0.920, *P* = 0.005), *phylum.Actinobacteria* (*OR* = 0.614, 95% *CI* 0.419–0.900, *P* = 0.012). Similar results were obtained in ML analyses. Nevertheless, these associations were no longer significant after the Bonferroni correction.

For EPTB, 195 bacterial taxa on the risk of EPTB were shown in the Additional file 2: Fig. S1 and Additional file 1: Table S8. As illustrated in Additional file 4: Fig. S3, the results of the IVW analysis showed that *class.Mollicutes* (*OR* = 1.669, 95% *CI* 1.026–2.716, *P* = 0.039), *family.Bacteroidaceae* (*OR* = 2.161, 95% *CI* 1.051–4.447, *P* = 0.036), *genus..Eubacteriumrectalegroup* (*OR* = 2.523, 95% *CI* 1.221–5.214, *P* = 0.012), *genus..Eubacteriumventriosumgroup* (*OR* = 1.765, 95% *CI* 1.058–2.943, *P* = 0.030), *genus.Bacteroides* (*OR* = 2.161, 95% *CI* 1.051–4.447, *P* = 0.036), *phylum.Tenericutes* (*OR* = 1.669, 95% *CI* 1.026–2.716, *P* = 0.039) significantly increased the risk of EPTB. In contrast, several bacterial taxa showed negative associations with EPTB risk, including *genus..Eubacteriumxylanophilumgroup* (*OR* = 0.567, 95% *CI* 0.323–0.995, *P* = 0.048), *genus.Barnesiella* (*OR* = 0.526, 95% *CI* 0.302–0.919, *P* = 0.024), *genus.Intestinimonas* (*OR* = 0.600, 95% *CI* 0.390–0.923, *P* = 0.020), *genus.LachnospiraceaeUCG001* (*OR* = 0.617, 95% *CI* 0.403–0.944, *P* = 0.026), *genus.Lactococcus* (*OR* = 0.669, 95% *CI* 0.448–0.999, *P* = 0.049), *genus.Marvinbryantia* (*OR* = 0.538, 95% *CI* 0.302–0.959, *P* = 0.035), *genus.Streptococcus* (*OR* = 0.485, 95% *CI* 0.265–0.886, *P* = 0.019). ML and weighted median methods yielded the similar direction for the casual effects of these bacterial taxa on the risk of EPTB. Nevertheless, these associations were no longer significant after the Bonferroni correction.

### Common bacterial taxa between UK Biobank database and FinnGen database

**Fig. 5:**
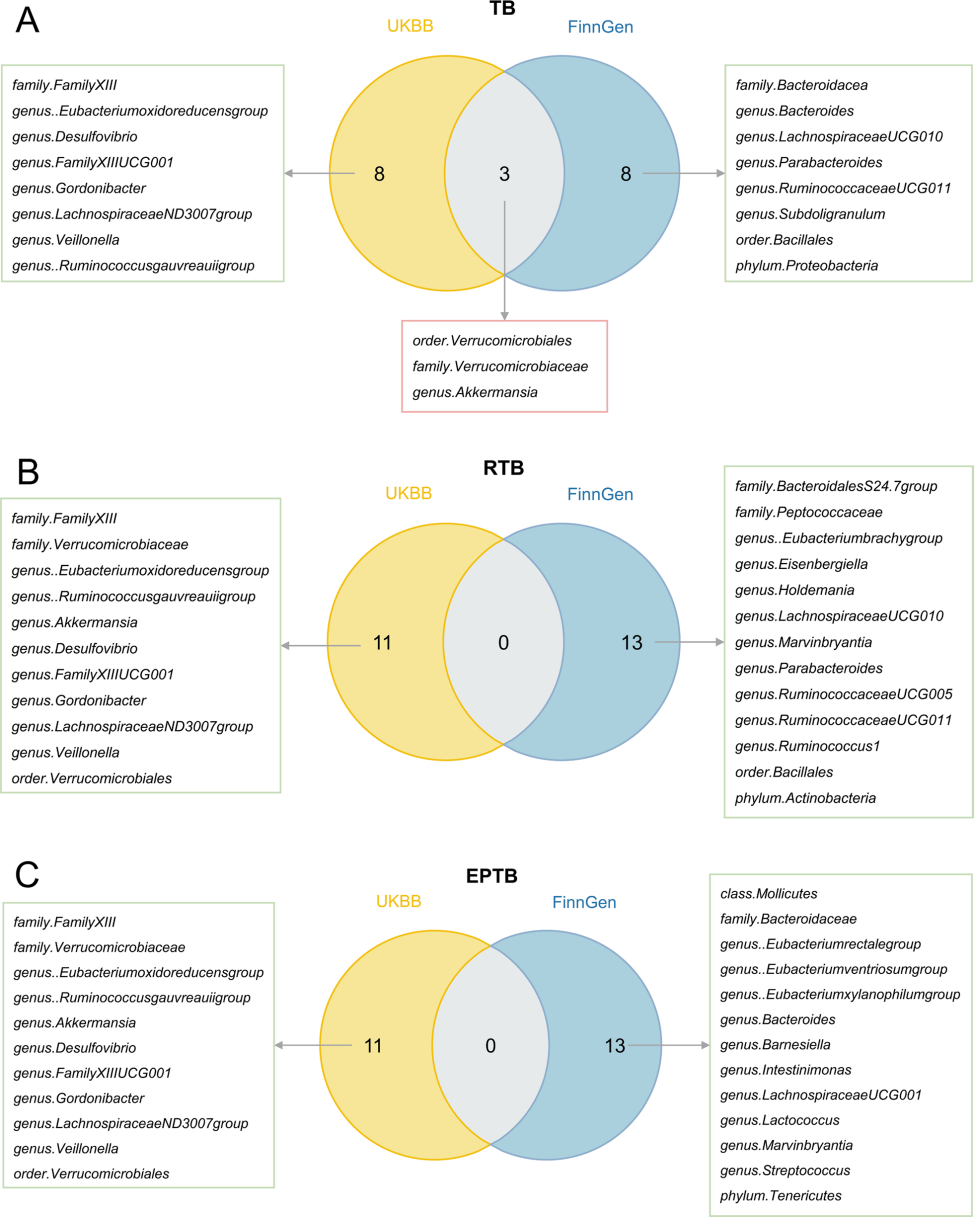
The common bacterial traits in two databases for TB, RTB, and EPTB. **A** The common bacterial traits in two databases for TB. **B** The common bacterial traits in relation to both TB and RTB in two databases. **C** The common bacterial traits in relation to both TB and EPTB in two databases. TB, tuberculosis; RTB, respiratory tuberculosis; EPTB, extrapulmonary tuberculosis; UKBB, the UK Biobank database; FinnGen, the FinnGen database

**Fig. 6:**
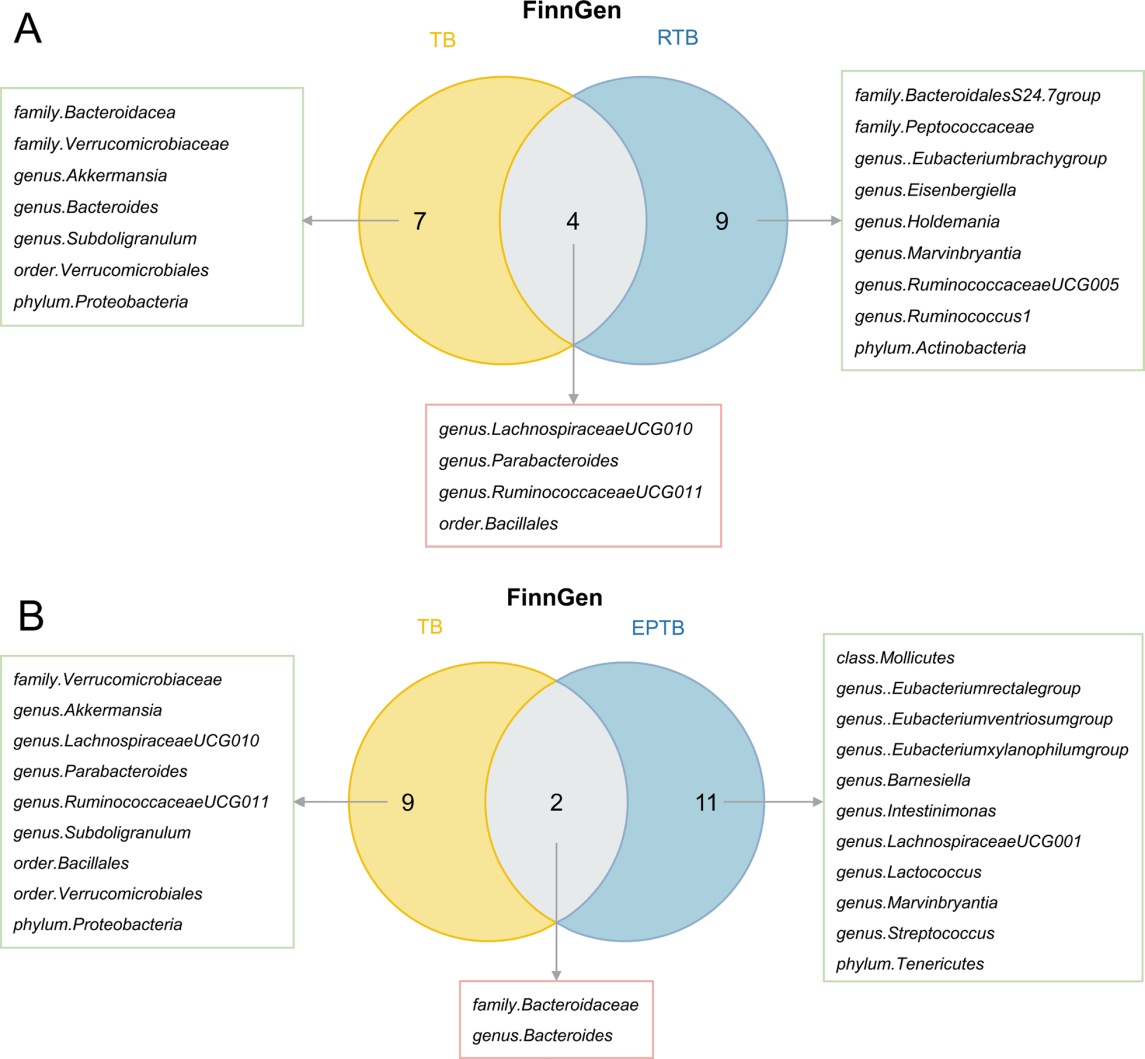
The common bacterial traits in the FinnGen database for RTB and EPTB. **A** The common bacterial traits in relation to both TB and RTB in the FinnGen database. **B** The common bacterial traits in relation to both TB and EPTB in the FinnGen database. TB, tuberculosis; RTB, respiratory tuberculosis; EPTB, extrapulmonary tuberculosis; FinnGen, the FinnGen database

Three bacterial taxa, including *genus.Akkermansia*, *family.Verrucomicrobiacea*, and *order.Verrucomicrobiales*, had a significant causal association with TB commonly in both databases. Notably, according to the results of MR analyses in the UK Biobank database, no bacterial taxa were identified to overlap with RTB or EPTB (Fig. 5). Compared with the analysis results of TB in the FinnGen database, *genus.LachnospiraceaeUCG010*, *genus.Parabacteroides*, *genus.RuminococcaceaeUCG011*, and *order.Bacillales* were common bacterial taxa of RTB, while *family.Bacteroidacea* and *genus.Bacteroides* were common bacterial taxa of EPTB (Fig. 6).

### Bi-directional causal inference between the gut microbiota and TB

To assess possible reverse causal associations, we applied reverse MR analysis with TB, RTB, or EPTB as exposure and gut microbiota as outcome. We identified 113 IVs for TB in the UK Biobank database, 83 IVs for TB in the FinnGen database, 77 IVs for RTB in the FinnGen database, 78 IVs for EPTB in the FinnGen database (Additional file 1: Table S9) and did not find any reverse causal correlation (Additional file 1: Table S10). We subsequently applied the MR Steiger directionality test to further confirm the true direction of causal association, and the result supported that gut microbiome was the influencing factor of TB (Additional file 1: Table S11).

### Sensitivity analysis

Finally, sensitivity analyses were performed for all the results (Additional file 1: Table S12, Additional file 1: Table S13). We found that no evidence of horizontal pleiotropic of exposure factors was detected when using the MR-Egger regression detection and the MR-PRESSO global test (*P* > 0.05). The results of Cochran’s IVW Q test showed no significant heterogeneity of IVs. Similarly, the leave-one-out sensitivity analyses indicated that no single SNP significantly affected the causal association (Additional file 5: Fig. S4, Additional file 6: Fig. S5, Additional file 7: Fig. S6, Additional file 8: Fig. S7).

## Discussion

Despite the success of chemotherapy over the past few decades, TB remains one of the leading infectious disease killers worldwide [1, 3]. In addition to some known influencing factors, including persistent global poverty, high HIV prevalence, drug resistance and etc. [40], the gut microbiota is an emerging host factor associated with TB. However, the causal association between gut microbiota and TB, as well as the direction of the causal association, remains poorly understood. In the present study, to the best of our knowledge, we first applied a two-sample MR analysis to assess the causal relationship between gut microbiota and TB by using multiple genetic databases. Concerning the UK Biobank database, our results indicated that 11 bacterial taxa had suggestive risk factors with TB and 3 of them were validated in the FinnGen database. We also identified 4 bacterial taxa associated with RTB and 2 bacterial taxa associated with EPTB in the FinnGen database. In reverse MR analysis, no causal association were identified. In short, these results emphasized the potential role of specific gut microbiota in the development and progression of different types of TB.

The taxonomic composition of the human gut microbiota is dominated by *Firmicutes*, *Bacteroidetes*, *Actinobacteria*, *Proteobacteria*, and other important phyla, including *Verrucomicrobia*, *Fusobacteria*, and *Euryarchaeota* [41]. *Genus.Akkermansia, family.Verrucomicrobiacea, *and *order.Verrucomicrobiales,* which were identified as risk factors for TB in our study, belong to the *Verrucomicrobia* phylum and are predominantly found within the epithelial layer of the human intestinal mucosa. *Akkermansia muciniphila* (*A.muciniphila*) is the sole representative species of *Verrucomicrobia* in the gut [42] and thus the relative abundance of the two may serve as an indicator for each other. Contrary to our findings, a cohort study conducted in China reported a significant decrease in the abundance of gut *A.muciniphila* of individuals with active TB compared to that of healthy subjects [43]. Although as a beneficial bacterium, *Verrucomicrobia* has been postulated that it may have a direct association with inflammation within the colon [44, 45]. For example, *A.muciniphila* is capable of degrading mucin and promoting gut inflammation [46]. In murine colonic tissues, the abundance of *Verrucomicrobia* also exhibited a discernible positive correlation with the expression of pro-inflammatory genes, such as TNF-α, NOS2, and IL-1β [47]. Elevated levels of gut inflammation are poised to disrupt the immune resistance against microorganisms. Considering the relatively limited research conducted on the *Verrucomicrobia* phylum in the context of TB, our results have important implications for enhancing understanding in this field.

In this study, we found that *genus.LachnospiraceaeUCG010*, *genus.Parabacteroides*, *genus.RuminococcaceaeUCG011*, and *order.Bacillales* were common traits in relation to both RTB and TB. *Lachnospiraceae* and *Ruminococcaceae* are crucial for development of the gut [48] and our results align with an earlier finding that the monkeys with enriched *Lachnospiraceae* were more susceptible to TB infection and the severe TB group exhibited an elevated abundance of *Ruminococcaceae* [49]. However, in prior population-observed investigations, the features of the two microbes were not consistent [14, 16]. Nevertheless, the bacteria from the two families were able to employ 7α-dehydroxylation to generate deoxycholic acid (DCA) and lithocholic acid (LCA) from cholic acid (CA) and chenodeoxycholic acid (CDCA), respectively. DCA and LCA, the common secondary bile acids, act as mediators of inflammation, influencing the body's immune balance [50] and heightening its vulnerability to pathogens. In a cross-sectional study, the higher abundance of *genus.Parabacteroides* in patients with untreated active TB [15] lends support to our finding. *Parabacteroides* is a gram-negative bacterium containing lipopolysaccharide, a potent endotoxin recognized for its capacity to induce a robust pro-inflammatory reaction within the host [51]. *Order.Bacillales* is a member of the *Firmicutes*, which is quite resistant to adverse external factors [52]. Our results supported *Order.Bacillales* as a probiotic with a potential beneficial role in TB treatment and immunization strategies.

Furthermore, the present study uncovered significant differences in the characteristics of gut microbial distribution between EPTB and RTB. *Family.Bacteroidacea* and *genus.Bacteroides* were identified as common traits in relation to both EPTB and TB. However, decreased *Bacteroidetes* has been observed in intestinal tuberculosis (ITB) and tuberculosis meningitis (TBM) [53-55]. Huang et al. [56] have indicated that dysbiosis caused by a reduced *Firmicutes* to *Bacteroidetes* ratio in TB infection was associated with systemic pro-inflammation. It is speculated that short-chain fatty acids (SCFAs) in the gastrointestinal tract may play a potential immunomodulatory role [17]. SCFAs are essential for maintaining a homeostatic environment, which are mainly produced by the phyla *Bacteroidetes* and *Firmicutes*. While the anti-inflammatory properties [57], it is noteworthy that evidences have also demonstrated their potential pro-inflammatory attributes [57, 58]. For instance, SCFAs have been found to facilitate the generation of reactive oxygen species (ROS) [57, 59], thus enhancing inflammatory response, potentially implicating the activation of intracellular inflammatory mechanisms (the inflammasome) [58]. EPTB may be more challenging to diagnose and treat [60], and our result would provide valuable insights for targeted therapeutic approaches.

This study made full use of publicly available comprehensive GWAS data for MR analysis, reducing the interference of confounding factors and false causal relationships, and ensuring the reliability of causal inferences. However, several limitations still exist in this study. First, across the spectrum from genes to phenotypes, a multitude of sources of variation exist, and we have striven to meticulously mitigate the confounding biases linked to these sources. Nonetheless, confounding factors endure, especially those that remain uncharacterized. Second, insufficiently stringent control thresholds were applied in the selection of IVs. Elevating the threshold helps to identify more potentially valuable IVs, and bidirectional MR analysis and sensitivity analysis further ensure the stability of the results. Finally, in this study, the gut microbiota data were sourced from MiBioGen consortium, and TB data were obtained from UK Biobank database and FinnGen database. Although these datasets are of high quality, it is important to note that there may be differences in the frequency distribution of these three databases that may introduce some potential biases. In the future, we hope our findings will be supported by larger data and evidence from in vitro experiments.

## Conclusion

In conclusion, through a bidirectional two-sample MR analysis, we comprehensively evaluated the causal-effect relationships between the gut microbiota and TB, RTB, and EPTB. Our current study is one that corroborates and enriches existing knowledge by providing evidence of genetic causation. The findings will provide crucial scientific evidence for the potential application of the gut microbiota as a preventive, diagnostic and therapeutic tool for TB.

### Supplementary Information

**Additional file 1: Table S1.** STROBE-MR Checklist. **Table S2.** Excluded single-nucleotide polymorphisms of potential confounders. **Table S3.** Single-nucleotide polymorphisms used as instrumental variables for TB in the UK Biobank database in MR analysis. **Table S4.** Single-nucleotide polymorphisms used as instrumental variables for TB in the FinnGen database in MR analysis. **Table S5.** Full results of MR estimates for the association between gut microbiota and TB in the UK Biobank database. **Table S6.** Full results of MR estimates for the association between gut microbiota and TB in the FinnGen database. **Table S7.** Full results of MR estimates for the association between gut microbiota and RTB in the FinnGen database. **Table S8.** Full results of MR estimates for the association between gut microbiota and EPTB in the FinnGen database. **Table S9.** Single-nucleotide polymorphisms used as instrumental variables for gut microbiota at the threshold of *P* < 1e−5 in reverse MR analysis. **Table S10.** MR results of causal links between TB on gut microbiota at the threshold of *P* < 1e−5 in the reverse MR analysis. **Table S11.** Results of MR Steiger direction test. **Table S12.** Results of sensitivity analyses between gut microbiota and TB with IVW *P* < 0.05. **Table S13.** Results of sensitivity analysEs between TB and gut microbiota in the reverse MR analysis.

**Additional file 2: Fig. S1.** Causal effect of the gut microbiota on RTB or EPTB by MR analyses in the FinnGen database. **A** Causal effect of the gut microbiota on RTB by MR analyses in the FinnGen database. **B** Causal effect of the gut microbiota on EPTB by MR analyses in the FinnGen database. From outside to inside, the P values of IVW_*p*, ML_*p*, MR Egger_*p*, WMe_*p*, and WMo_*p* were represented, respectively. RTB, respiratory tuberculosis; EPTB, extrapulmonary tuberculosis; IVW, inverse variance weighted; ML, maximum likelihood; WMe, weighted median; WMo, weighted mode.

**Additional file 3: Fig. S2.** A forest plot of causal effect of gut microbiota on RTB in the FinnGen database (*P-*_*IVW*_ < 0.05). RTB, respiratory tuberculosis; OR, odds ratio; CI, confidence interval.

**Additional file 4: Fig. S3.** A forest plot of causal effect of gut microbiota on EPTB in the FinnGen database (*P-*_*IVW*_ < 0.05). EPTB, extrapulmonary tuberculosis; OR, odds ratio; CI, confidence interval.

**Additional file 5: Fig. S4.** Leave-one-out plots for the causal effect of gut microbiota on TB in the UK Biobank database. TB, tuberculosis. **A** *Family.FamilyXIII*. **B** *Family.Verrucomicrobiaceae*. **C** *Genus..Eubacteriumoxidoreducensgroup*. **D** *Genus..Ruminococcusgauvreauiigroup*. **E** *Genus.Akkermansia*. **F** *Genus.Desulfovibrio*. **G** *Genus.FamilyXIIIUCG001*. **H** *Genus.Gordonibacter*. **I** *Genus.LachnospiraceaeND3007group*. **J** *Genus.Veillonella*. **K** *Order.Verrucomicrobiales*.

**Additional file 6: Fig. S5.** Leave-one-out plots for the causal effect of gut microbiota on TB in the FinnGen database. TB, tuberculosis. **A** *Family.Bacteroidaceae*. **B** *Family.Verrucomicrobiaceae*. **C** *Genus.Akkermansia*. **D** *Genus.Bacteroides*. **E** *Genus.LachnospiraceaeUCG010*. **F** *Genus.Parabacteroides*. **G** *Genus.RuminococcaceaeUCG011*. **H** *Genus.Subdoligranulum*. **I** *Order.Bacillales*. **J** *Order.Verrucomicrobiales*. **K** *Phylum.Proteobacteria*.

**Additional file 7: Fig. S6.** Leave-one-out plots for the causal effect of gut microbiota on RTB in the FinnGen database. RTB, respiratory tuberculosis. **A** *Family.BacteroidalesS24.7group*. **B** *Family.Peptococcaceae*. **C** *Genus..Eubacteriumbrachygroup*. **D** *Genus.Eisenbergiella*. **E** *Genus.Holdemania*. **F** *Genus.LachnospiraceaeUCG010*. **G** *Genus.Marvinbryantia*. **H** *Genus.Parabacteroides*. **I** *Genus.RuminococcaceaeUCG005*. **J** *Genus.RuminococcaceaeUCG011*. **K** *Genus.Ruminococcus1*. **L** *Order.Bacillales*. **M** *Phylum.Actinobacteria*.

**Additional file 8: Fig. S7.** Leave-one-out plots for the causal effect of gut microbiota on EPTB in the FinnGen database. EPTB, extrapulmonary tuberculosis. **A** *Class.Mollicutes*. **B** *Family.Bacteroidaceae*. **C** *Genus..Eubacteriumrectalegroup*. **D** *Genus..Eubacteriumventriosumgroup*. **E** *Genus..Eubacteriumxylanophilumgroup*. **F** *Genus.Bacteroides*. **G** *Genus.Barnesiella*. **H** *Genus.Intestinimonas*. **I** *Genus.LachnospiraceaeUCG001*. **J** *Genus.Lactococcus*. **K** *Genus.Marvinbryantia*. **L** *Genus.Streptococcus*. **M** *Phylum.Tenericutes*.

## Data Availability

The datasets analyzed in the current study are available in the MiBioGen, https://mibiogen.gcc.rug.nl/menu/main/home, the GWAS catalog, https://www.ebi.ac.uk/gwas/studies/GCST90038710, and the FinnGen consortium, https://www.finngen.fi/en.

## Abbreviations

TB: Tuberculosis
RTB: Respiratory tuberculosis
EPTB: Extrapulmonary tuberculosis
MR: Mendelian randomization
GWAS: Genome-wide association study
IVW: Inverse variance weighted
ML: Maximum likelihood
IVs: Instrumental variables
*M.tuberculosis*: *Mycobacterium tuberculosis*
SNPs: Single nucleotide polymorphisms
MAF: Minor allele frequency
EAF: Effect allele frequency
MR-PRESSO: Mendelian randomization pleiotropy residual sum and outlier
OR: Odds ratio
CI: Confidence interval
BMI: Body mass index
DNA: Deoxyribonucleic acid
*A.muciniphila*: *Akkermansia muciniphila*
DCA: Deoxycholic acid
LCA: Lithocholic acid
CA: Cholic acid
CDCA: Chenodeoxycholic acid
ITB: Intestinal tuberculosis
TBM: Tuberculosis meningitis
SCFAs: Short-chain fatty acids
ROS: Oxygen species
