# Supplementary figures and images for: Causal relationship between gut microbiota and tuberculosis: a bidirectional two-sample Mendelian randomization analysis
Source: Respir Res. 2024 Jan 4;25:16. doi: 10.1186/s12931-023-02652-7 (PMC10765819; doi:10.1186/s12931-023-02652-7)

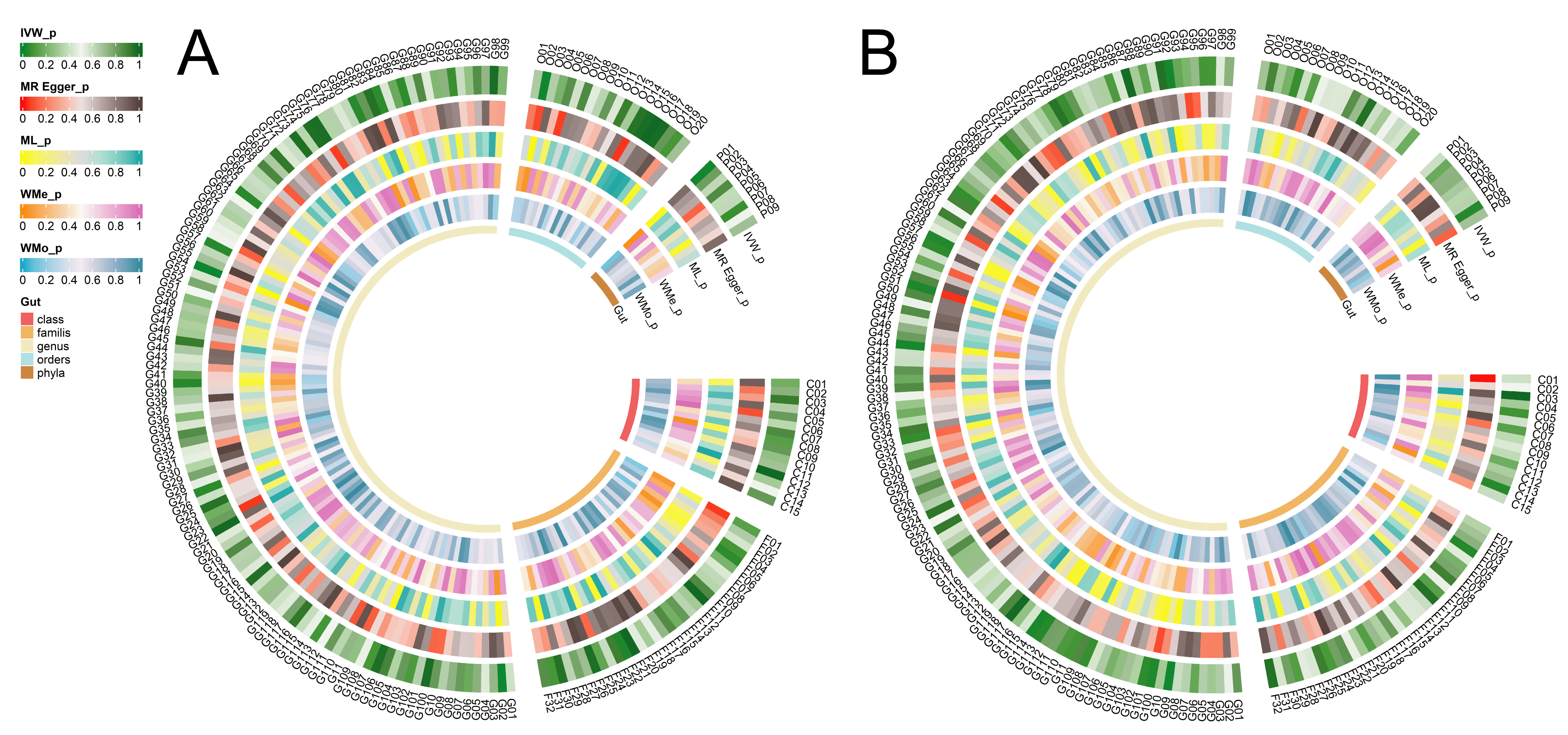

Supplement: Supplementary file 2 — Additional file 2: Fig. S1. Causal effect of the gut microbiota on RTB or EPTB by MR analyses in the FinnGen database. A Causal effect of the gut microbiota on RTB by MR analyses in the FinnGen database. B Causal effect of the gut microbiota on EPTB by MR analyses in the FinnGen database. From outside to inside, the P values of IVW_p, ML_p, MR Egger_p, WMe_p, and WMo_p were represented, respectively. RTB, respiratory tuberculosis; EPTB, extrapulmonary tuberculosis; IVW, inverse variance weighted; ML, maximum likelihood; WMe, weighted median; WMo, weighted mode. [file 12931_2023_2652_MOESM2_ESM.jpg]

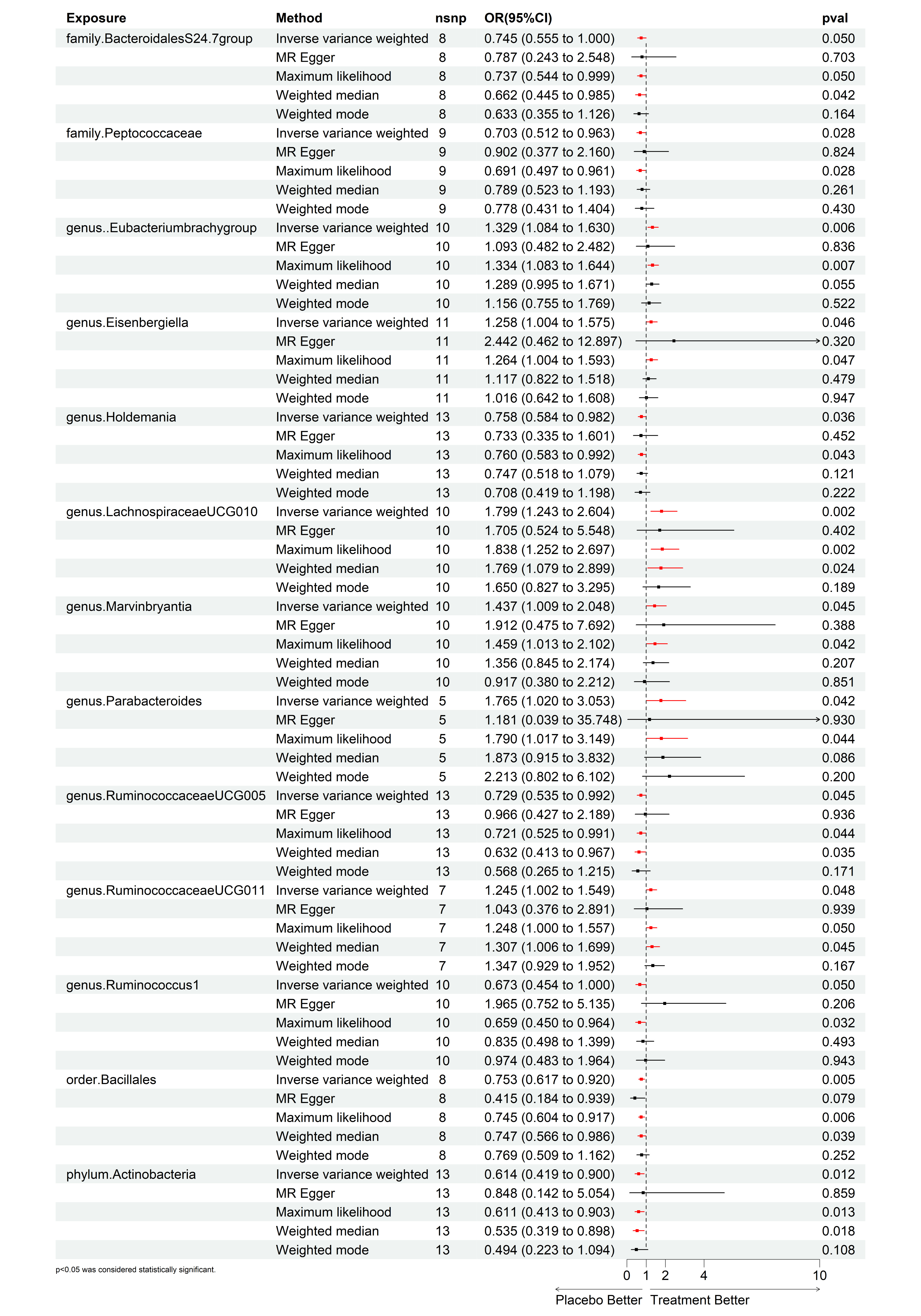

Supplement: Supplementary file 3 — Additional file 3: Fig. S2. A forest plot of causal effect of gut microbiota on RTB in the FinnGen database (P-IVW < 0.05). RTB, respiratory tuberculosis; OR, odds ratio; CI, confidence interval. [file 12931_2023_2652_MOESM3_ESM.jpg]

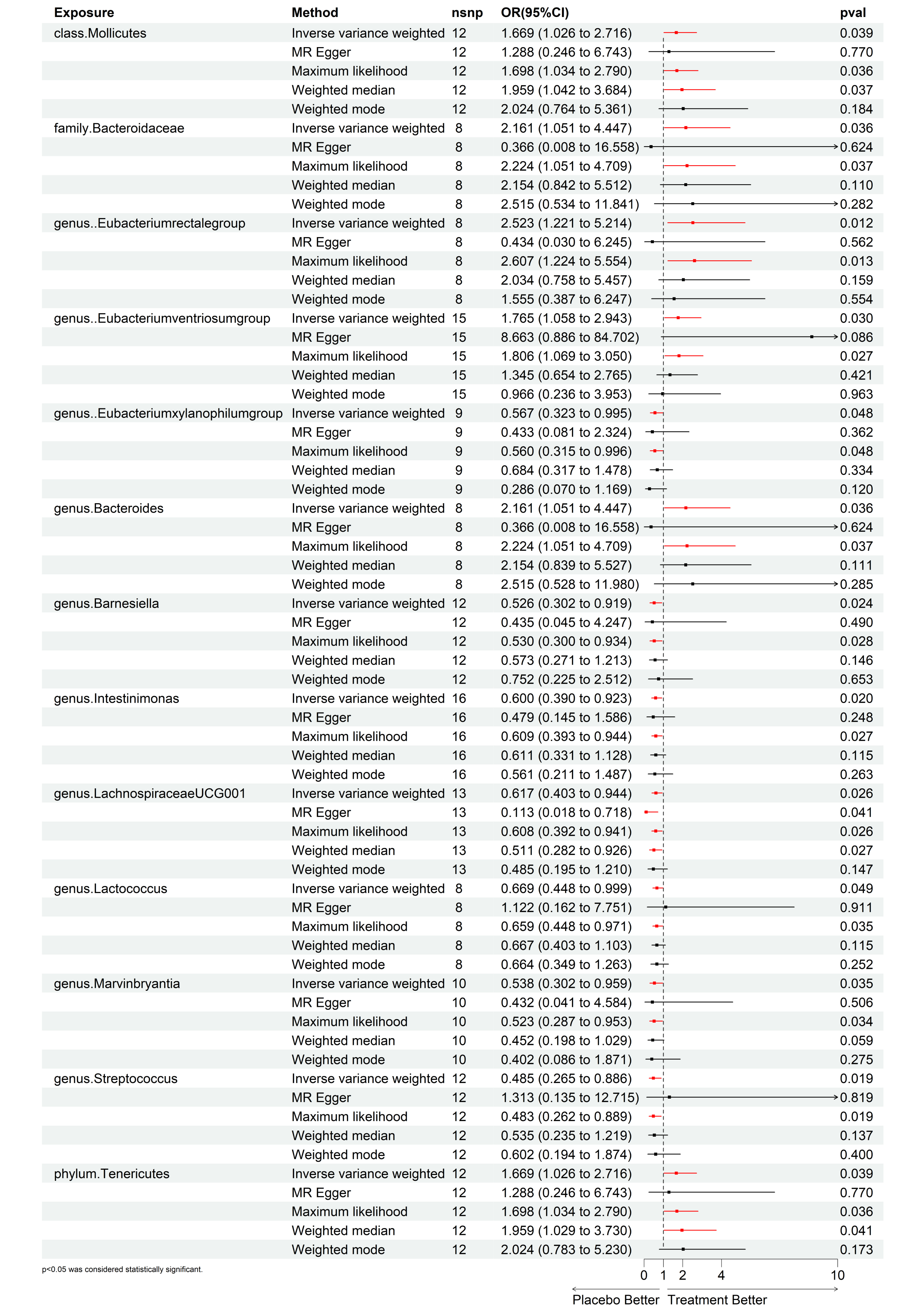

Supplement: Supplementary file 4 — Additional file 4: Fig. S3. A forest plot of causal effect of gut microbiota on EPTB in the FinnGen database (P-IVW < 0.05). EPTB, extrapulmonary tuberculosis; OR, odds ratio; CI, confidence interval. [file 12931_2023_2652_MOESM4_ESM.jpg]
